# Febuxostat pretreatment attenuates myocardial ischemia/reperfusion injury via mitochondrial apoptosis

**DOI:** 10.1186/s12967-015-0578-x

**Published:** 2015-07-02

**Authors:** Shulin Wang, Yunpeng Li, Xudong Song, Xianbao Wang, Cong Zhao, Aihua Chen, Pingzhen Yang

**Affiliations:** Department of Cardiology, Zhujiang Hospital of Southern Medical University, No. 253, Gongye Road, Guangzhou, 510280 China

**Keywords:** Febuxostat, Myocardial ischemia/reperfusion injury, Mitochondrial damage, Apoptosis

## Abstract

**Background:**

Febuxostat is a selective inhibitor of xanthine oxidase (XO). XO is a critical source of reactive oxygen species (ROS) during myocardial ischemia/reperfusion (I/R) injury. Inhibition of XO is therapeutically effective in I/R injury. Evidence suggests that febuxostat exerts antioxidant effects by directly scavenging ROS. The present study was performed to investigate the effects of febuxostat on myocardial I/R injury and its underlying mechanisms.

**Methods:**

We utilized an in vivo mouse model of myocardial I/R injury and an in vitro neonatal rat cardiomyocyte (NRC) model of hypoxia/reoxygenation (H/R) injury. Mice were randomized into five groups: Sham, I/R (I/R + Vehicle), I/R + FEB (I/R + febuxostat), AL + I/R (I/R + allopurinol) and FEB (febuxostat), respectively. The I/R + FEB mice were pretreated with febuxostat (5 mg/kg; i.p.) 24 and 1 h prior to I/R. NRCs received febuxostat (1 and 10 µM) at 24 and 1 h before exposure to hypoxia for 3 h followed by reoxygenation for 3 h. Cardiac function, myocardial infarct size, serum levels of creatine kinase (CK) and lactate dehydrogenase (LDH), and myocardial apoptotic index (AI) were measured in order to ascertain the effects of febuxostat on myocardial I/R injury. Hypoxia/reperfusion (H/R) injury in NRCs was examined using MTT, LDH leakage assay and terminal deoxynucleotidyl transferase dUTP nick end labeling (TUNEL) assay. The underlying mechanisms were determined by measuring ROS production, mitochondrial membrane potential (ΔΨm), and expression of cytochrome c, cleaved caspases as well as Bcl-2 protein levels.

**Results:**

Myocardial I/R led to an elevation in the myocardial infarct size, serum levels of CK and LDH, cell death and AI. Furthermore, I/R reduced cardiac function. These changes were significantly attenuated by pretreatment with febuxostat and allopurinol, especially by febuxostat. Febuxostat also protected the mitochondrial structure following myocardial I/R, inhibited H/R-induced ROS generation, stabilized the ΔΨm, alleviated cytosolic translocation of mitochondrial cytochrome C, inhibited activation of caspase-3 and -9, upregulated antiapoptotic proteins and downregulated proapoptotic proteins.

**Conclusions:**

This study revealed that febuxostat pretreatment mediates the cardioprotective effects against I/R and H/R injury by inhibiting mitochondrial-dependent apoptosis.

## Background

Myocardial ischemia/reperfusion (I/R) injury is characterized by altered metabolic disorders and structural damage during reperfusion following myocardial ischemia [1]. The myocardial injury paradoxically reduces the benefit of procedures, such as thrombolytic therapy, percutaneous coronary intervention, and coronary bypass surgery [2]. Therefore, amelioration of ischemia- and reperfusion-induced myocardial injury is a clinical imperative.

An increase in reactive oxygen species (ROS) production is one of the key events in I/R injury [3]. Excessive ROS generation leads to mitochondrial injury, including loss of mitochondrial membrane potential (ΔΨm) and results in a series of events, particularly apoptosis [4]. Consequently, inhibition of ROS production and protection of mitochondria from oxidative damage are effective strategies to ameliorate myocardial I/R injury. Xanthine oxidase (XO) is involved in the generation of O^2−^ in response to hypoxia. During reperfusion, XO is also a critical source of ROS [5]. Febuxostat, a non-purine selective XO inhibitor, has been shown to have beneficial effects in renal I/R injury [6], suggesting that febuxostat reduced oxidative stress and suppressed apoptosis. However, the effect of febuxostat pretreatment on ischemia- and reperfusion-induced myocardial injury via mitochondrial apoptosis remains unclear.

Therefore, the objectives of the present study were: (1) to determine if febuxostat exerts cardioprotective effects in I/R injury and neonatal rat cardiomyocytes (NRCs) subjected to H/R injury; and (2) to investigate the mechanisms underlying the cardioprotective effects of febuxostat.

## Methods

### Materials and reagents

Male C57BL/6 mice (20–25 g) were obtained from the Laboratory Animal Center of Guangdong Province. One hundred eight mice were included in the study. Febuxostat was purchased from Teijin (Tokyo, Japan), and diluted in 0.5% methylcellulose (Sigma, St. Louis, MO, USA). Dulbecco Modified Eagle’s Medium (DEME), 5-bromo-2-deoxyuridine (BrdU), fetal bovine serum (FBS), 2,3,5-triphenyltetrazolium chloride (TTC) and Evan’s Blue dye were purchased from Sigma-Aldrich (St. Louis, MO, USA). Serum creatine kinase (CK) and lactate dehydrogenase (LDH) commercial kits were obtained from Biovision (Mountain View, CA, USA). Caspase-3 and caspase-9 activity were measured using commercial kits (Biovision, Mountain View, CA, USA). The Hoechst staining kit was obtained from Promega (Beijing, China). ROS and Mitochondrial Membrane Potential Assay Kits were obtained from Beyotime (Jiangsu, China). Antibodies against Bcl-2 (1:1,000), Bcl-X_L_ (1:1,000), Bax (1:1,000), Bak (1:1,000), Cytochrome C (1:1,000), Caspase-9 (1:1,000), Caspase-3 (1:1,000), and GAPDH were purchased from Cell Signaling Technology (Beverly, MA, USA). COX IV (1:1,000) was from Bioworld Corporation (Dublin, USA).

### Experimental animals and myocardial I/R model

All animals received humane care in accordance with the Guide for the Care and Use of Laboratory Animals published by the United States National Institute of Health (NIH Publication No. 85-23, revised 1996). All investigations were approved by the Bioethics Committee of Southern Medical University, Guangzhou, China. The I/R model was developed as described previously [7, 8]. Briefly, mice were anesthetized by an intraperitoneal injection of ketamine and pentobarbital sodium, and connected to a rodent ventilator. The left anterior descending (LAD) coronary artery was surgically ligated by passing a 7–0 silk suture under the LAD. Regional ischemia was confirmed by visual inspection of pale color of the myocardium and ST segment elevation on electrocardiogram. Animals in the Sham and FEB group were also anesthetized and a suture was passed under the LAD, without occlusion.

### Experimental protocols in vivo

Mice were randomized into three groups: (1) Sham, which received sham operation without coronary artery ligation and used as normal control; (2) I/R (I/R + Vehicle), which were pretreated with 0.5 ml of vehicle (0.5% methylcellulose); (3) I/R + FEB (I/R + febuxostat), the mice were pretreated with febuxostat (5 mg/kg) [9] in 0.5 ml methylcellulose; (4) ALL + I/R (I/R + allopurinol), allopurinol (30 mg/kg) in 0.5 ml methylcellulose; and (5) FEB (febuxostat), which received sham operation without coronary artery ligation. All pretreatments were administered through intraperitoneal injection 24 and 1 h before ischemia induction. Groups 2, 3, 4 were subjected to myocardial ischemia for 45 min, followed by reperfusion for 2 h. Following reperfusion, a portion of the blood was collected by cardiopuncture, and the heart was harvested and washed with ice-cold normal saline.

### In vitro studies

Neonatal rat cardiomyocytes (NRCs) were prepared from newborn (1- to 2-day old) Sprague–Dawley rats as previously described [10]. Briefly, ventricles of the newborn rats were isolated aseptically, and then digested using trypsin and collagenase, and purified by differential pre-plating. The resuspended cells were maintained in DMEM with 10% (v/v) fetal bovine serum, BrdU (100 μmol/L), followed by transfer to a culture dish under conditions of 95% atmosphere and 5% CO_2_ at a temperature of 37°C. Hypoxia/reoxygenation (H/R) was performed in vitro as described previously [11, 12]. The in vitro model of H/R included hypoxia for 3 h, and oxygenation for 3 h. The following groups were tested. (1) Control group: cells were cultured in a standard incubator (95% O_2_, 5% CO_2_, 37°C); (2) H/R: cells were incubated in the hypoxic chamber for 3 h (95% N_2_, 5% CO_2_, 37°C), and then reoxygenated in a standard incubator for 3 h (95% O_2_, 5% CO_2_, 37°C); (3) Vehicle (H/R + Veh): cells were cultured with methylcellulose 24 and 1 h prior to H/R; (4, 5) H/R + Feb: cells were exposed to febuxostat (1 and 10 µM) [9] at 24 and 1 h respectively prior to H/R; and (6) H/R + All: cells were administered allopurinol (10 µM) before hypoxia induction. (7) Feb: cells were exposed to febuxostat 10 µM at 24 and 1 h in a standard incubator (95% O_2_, 5% CO_2_, 37°C).

### Assessment of myocardial infarct size

Evan’s Blue-triphenyltetrazolium chloride (TTC) double staining methods were used to determine myocardial infarct size as described previously [13]. Following reperfusion for 2 h, the LAD was re-occluded and 0.2–0.3 ml of 2% solution of Evan’s Blue dye was injected into the right jugular vein to identify the area prone to ischemic damage, termed area at risk (AAR). When the right side of the heart turned blue, the heart was rapidly excised and rinsed in normal saline. The left ventricle (LV) was isolated and frozen at −20°C for 30 min. The LV was then cut into five 1-mm thick slices, which were incubated in 1% TTC for 15 min at 37.0°C. The infarct area (INF; white) and the area at risk (AAR; red and white) from each segment were measured using an image analyzer. Ratios of area at risk vs. left ventricle (AAR/LV), infarct area vs. area at risk (INF/AAR) and infarct area vs. left ventricle (INF/LV) were calculated.

### Echocardiography

Echocardiography was performed 1 week before and after I/R induction. An echocardiography system with a Sonos 4500 and a 15–16 MHz transducer (Philips Corporation) was used. Transthoracic echocardiography was performed to obtain both 2-dimensional and M-mode images. To determine cardiac structure and function, left ventricular end diastolic dimension (LVEDD), left ventricular end systolic dimension (LVESD), left ventricular ejection fraction (LVEF), and fractional shortening (FS%) were analyzed from images as previously described [8, 14]. FS% was calculated as (LVEDD − LVESD)/LVEDD × 100%.

### Serum creatine kinase (CK) and lactate dehydrogenase (LDH) levels

In order to determine the degree of myocardial injury, the serum myocardial enzymes LDH and CK were measured using commercial kit reagents according to the manufacturers’ instructions.

### TUNEL assay and assessment of caspases activity

Myocardial apoptosis was assessed using DeadEndTM Fluorometric terminal deoxynucleotidyl-transferase dUTP nick-end labeling (TUNEL) assay. The total cardiomyocyte nuclei were identified by Hoechst 33258, and apoptotic nuclei were labeled with green fluorescein dye. In order to examine caspase-3 and caspase-9 activity, we used commercial kit reagents with procedures outlined by the manufacturer (Biovision, Mountain View, CA). Samples of whole left ventricular homogenate were prepared and tissues were homogenized in the cell lysis buffer followed by centrifugation. The supernatant was collected and used for the assay. Protein concentration in the lysate was measured and 100 µg lysate protein was used in 50 µl Cell Lysis Buffer for each assay. Caspase activity was monitored using a Microplate Reader at 405 nm.

### Cell viability

Cell viability was determined by the MTT assay, with 1 × 10^4^ cells/well seeded into 96-well plates. Following the experimental interventions, MTT solution was added into each well (5 mg/ml) and the plates were incubated for 4 h at 37°C. The viability was then measured by evaluating the absorbance at 570 nm. An LDH kit (Sigma) was used to measure the extent of cellular injury as previously described [15].

### Determination of NRCs apoptosis by TUNEL

NRCs apoptosis was assessed using the DeadEnd™ Fluorometric TUNEL System according to the manufacturer’s protocol. Cells were fixed with 4.0% formaldehyde in PBS for 25 min at 4°C, and incubated with 10 μM Hoechst 33258 for 15 min. Cells were observed under fluorescence microscopy.

### Measurement of ROS and Mitochondrial membrane potential (ΔΨm)

ROS and Mitochondrial Membrane Potential Assay Kits (S0033 and C2006) were used to measure ROS and ΔΨm of NRCs according to the manufacturer’s instructions. Techniques to measure ROS were performed as previously described [16]. Briefly, cells were incubated with the 1:1000 ROS-sensitive dye 2′,7′-dichloruorescein-diacetate (DCFH-DA) dilution, and then incubated for 20 min at 37°C. To measure ΔΨm, cells were seeded into laser confocal petri dishes. After treatment, the dishes were incubated with JC-1 staining solution (5 μg/ml) in an incubator for 20 min at 37°C. The cells were then washed twice with JC-1 staining buffer and confocal laser scanning microscopy (OLYMPUS FV1000) was used for detection.

### Electron transmission microscopy

After reperfusion for 2 h, the LVs were harvested, cut into 1-mm^3^ sections on ice and fixed with 2.5% glutaraldehyde. Osmium tetroxide (1% in 0.1 mol/l cacodylate) was used for post-fixation. Tissues were dehydrated with a series of ethanol rinses. Samples were embedded and sliced. The slices were stained and observed using electron transmission microscopy (PHILIPS CM10, Holland). Mitochondrial area was measured using the Scanning Probe Imaging Processor. In each specimen, the shape parameters of 30 mitochondria were measured.

### Western blot

Protein extracts from NRCs were subjected to Western blot. Protein concentrations were measured with a BCA Protein Assay Kit. Equal amounts of protein were loaded into lanes and were separated using SDS-PAGE, followed by transfer to a polyvinylidene fluoride membrane. The membranes were then blocked with 5% skim milk solution, followed by overnight incubation at 4°C with the appropriate primary antibody. The membranes were probed the following day with secondary antibodies for 1 h at room temperature, and then washed with Tris-buffered saline/0.1% Tween-20. The signals were detected using an electrochemiluminesence (ECL) system and scanned. The relative intensity of the bands was quantified using the Image J 3.0 system.

### Statistical analyses

Data were expressed as mean ± standard deviation (SD). One-way analysis of variance (ANOVA) followed by either a Bonferroni post hoc test or Student’s t test was used for statistical significance of multiple treatments as appropriate. A value of *P* < 0.05 was considered statistically significant.

## Results

### Effect of febuxostat on myocardial injury in vivo

**Figure 1:**
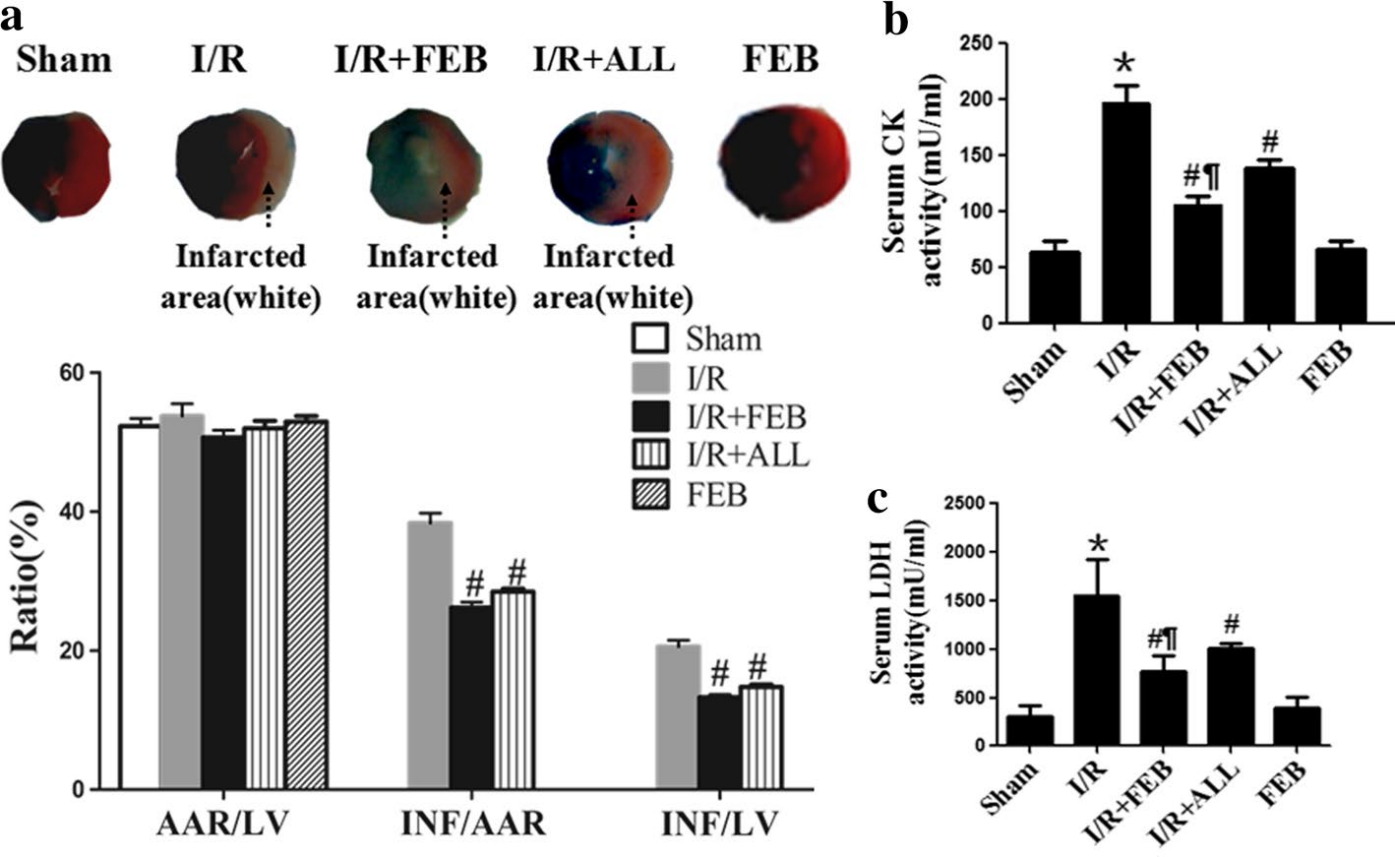
Effects of febuxostat on infarct size and necrosis in mice. **a** Representative images of myocardial infarct size and the ratio of AAR/LV, INF/AAR, and INF/LV. **b** Serum CK levels. **c** Serum LDH levels. The results were expressed as mean ± SD. (n = 7). *P < 0.01 vs. Sham, ^#^ *P* < 0.01, vs. I/R, ^¶^ *P* < 0.01 vs. I/R + ALL.

Figure 1 illustrates the effect of febuxostat treatment on infarct size and necrosis in mice. Representative myocardial infarct size images are shown in Figure 1a. Compared with Sham, I/R induced a significant increase in INF/AAR (INF/AAR: 38.3 ± 3.8 vs. 0; INF/LV: 20.5 ± 2.4 vs. 0; *P* < 0.01, respectively). However, infarct size in the I/R + FEB and I/R + ALL groups was markedly reduced compared with I/R (*P* < 0.01). There was no difference in AAR/LV between groups. The difference between the sham and FEB groups was not significant (*P* > 0.05).

Figure 1b, c shows the effect of febuxostat on serum CK and LDH activities. In comparison with Sham group, I/R treatment increased serum CK and LDH activities (*P* < 0.01). Febuxostat treatment effectively blocked the increase of CK and LDH activities compared with I/R mice (CK: 121.9 ± 16.6 vs. 196.3 ± 15.3 mU/ml; LDH: 765.5 ± 166.1 vs. 1549.8 ± 365.8 mU/ml; *P* < 0.01, respectively), similar to allopurinol (CK: 138.2 ± 7.18 vs. 196.3 ± 15.3 mU/ml; LDH: 1005.9 ± 53.1 vs. 1549.8 ± 365.8 mU/ml; *P* < 0.01, respectively). Compared with I/R + ALL mice, I/R + FEB markedly decreased CK and LDH activities (*P* < 0.05). There was no significant difference between the Sham group and FEB group (*P* > 0.05).

**Figure 2:**
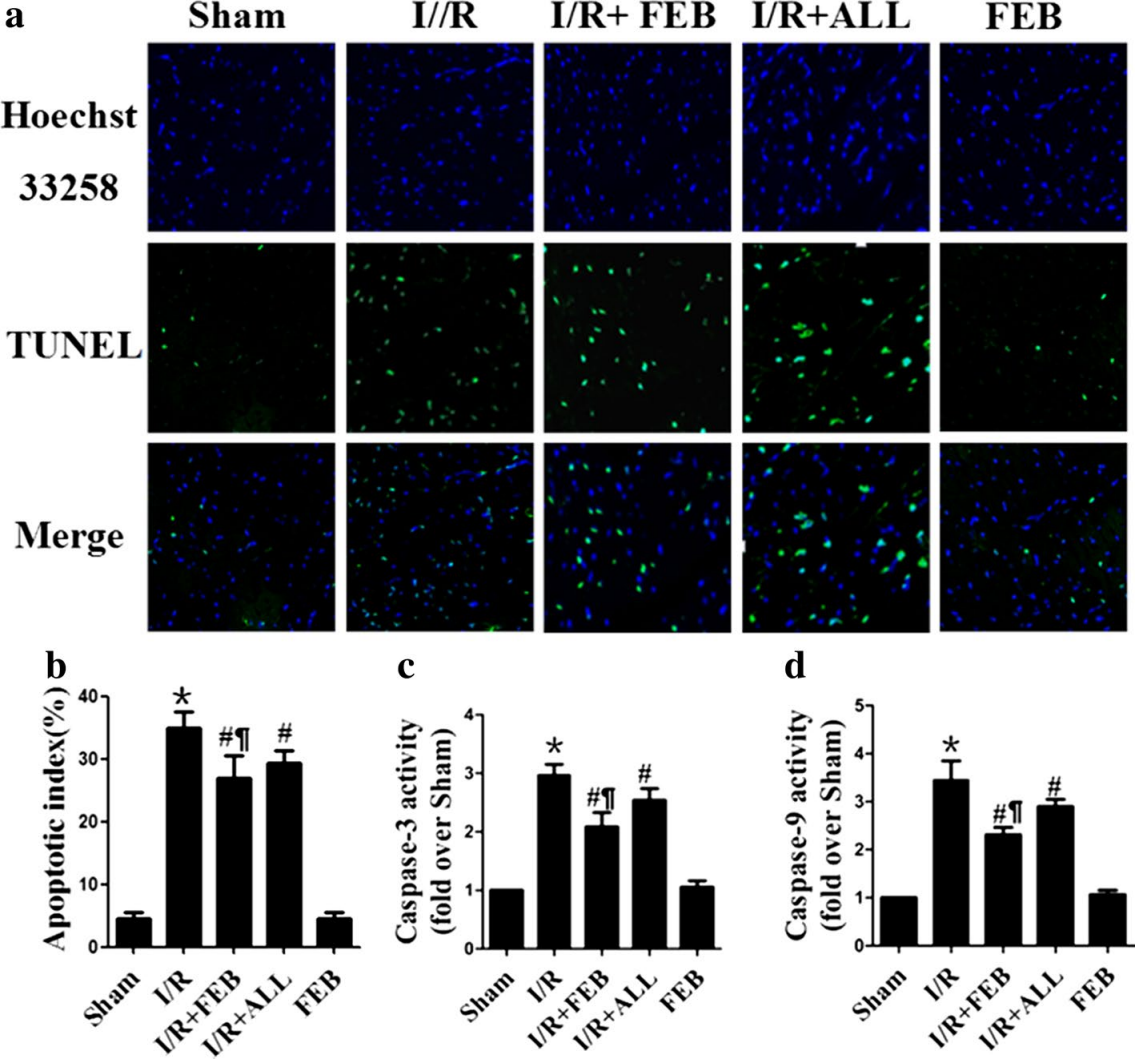
Effects of febuxostat on apoptosis in mice. **a** Representative photomicrographs (×200) of TUNEL staining in myocardial I/R tissue. *Green fluorescence* indicates TUNEL-positive apoptotic nuclei; *blue fluorescence* indicates total cardiomyocyte nuclei. **b** Apoptotic index. **c** Myocardial caspase-3 activity. **d** Myocardial caspase-9 activity. The results were expressed as mean ± SD (n = 7). The results were expressed as mean ± SD (n = 7). *P < 0.01 vs. Sham, ^#^ *P* < 0.01, vs. I/R, ^¶^ *P* < 0.01 vs. I/R + ALL.

Apoptosis is the major mechanism of cell death following I/R injury [17]. TUNEL and caspases activities were used to assess the level of apoptosis. Figure 2 illustrates the effect of febuxostat treatment on apoptosis in mice. In comparison with Sham group, I/R treatment increased the apoptotic index (34.9 ± 2.6 vs. 4.5 ± 0.9%, *P* < 0.01). Pretreatment with either febuxostat or allopurinol effectively attenuated the increase in apoptotic index (Febuxostat: 26.9 ± 3.7 vs. 34.9 ± 2.6%; Allopurinol: 29.4 ± 1.9 vs. 34.9 ± 2.6%, *P* < 0.01, respectively). Compared with I/R + ALL mice, I/R +FEB markedly decreased in apoptotic index (*P* < 0.01). Caspase-3 and -9 activities were increased in the myocardium following I/R compared to Sham (Caspase-3: 2.9 ± 0.2 vs. 1; Caspase-9: 3.5 ± 0.4 vs. 1, *P* < 0.01, respectively). The Caspase-3 and -9 activities in the febuxostat and allopurinol-pretreated groups was significantly lower than that in I/R group (*P* < 0.01). In comparison with I/R + ALL, I/R + FEB markedly decreased the Caspase-3 and -9 activities (Caspase-3: 2.1 ± 0.2 vs. 2.5 ± 0.2; Caspase-9: 2.2 ± 0.1 vs. 2.9 ± 0.2, *P* < 0.01, respectively). There was no significant difference between the Sham and FEB groups in apoptotic index and Caspases activities (*P* > 0.05).

### Effect of febuxostat on cardiac function

Echocardiograms were obtained after 1 week in order to determine the effect of febuxostat on cardiac function following I/R. Seven mice were enrolled in each group. I/R-induced reduction in cardiac function was significantly restored by pretreatment with febuxostat and allopurinol (*P* < 0.01, respectively) (Table 1).

**Table 1:** Effects of febuxostat on left ventricular parameters post-I/R myocardial injury

	Baseline			7-day post I/R		
	I/R (n = 7)	I/R + FEB (n = 7)	I/R + ALL (n = 7)	I/R (n = 7)	I/R + FEB (n = 7)	I/R + ALL (n = 7)
LVEDD (mm)	3.46 ± 0.05	3.46 ± 0.04	3.47 ± 0.04	3.97 ± 0.11	3.94 ± 0.08	3.96 ± 0.10
LVESD (mm)	2.26 ± 0.02	2.27 ± 0.02	2.25 ± 0.02	2.92 ± 0.07	2.74 ± 0.07^#^	2.80 ± 0.09^#^
FS (%)	34.6 ± 0.01	34.47 ± 0.01	35.27 ± 0.03	26.43 ± 0.03	30.39 ± 0.02^#^	28.77 ± 0.05^#^
EF (%)	66.6 ± 0.98	65.91 ± 1.42	65.16 ± 1.22	37.66 ± 1.30	41.93 ± 1.52^#^	39.73 ± 4.08^#^

Values are mean ± SD.

*LVEDD* left ventricular end-diastolic diameter, *LVESD* left ventricular end-systolic diameter, *FS* fractional shortening, *EF* ejection fraction, *I*/*R* ischemia/reperfusion.

^#^ *P* < 0.05 vs. I/R.

### Effect of febuxostat on H/R injury in NRCs

**Figure 3:**
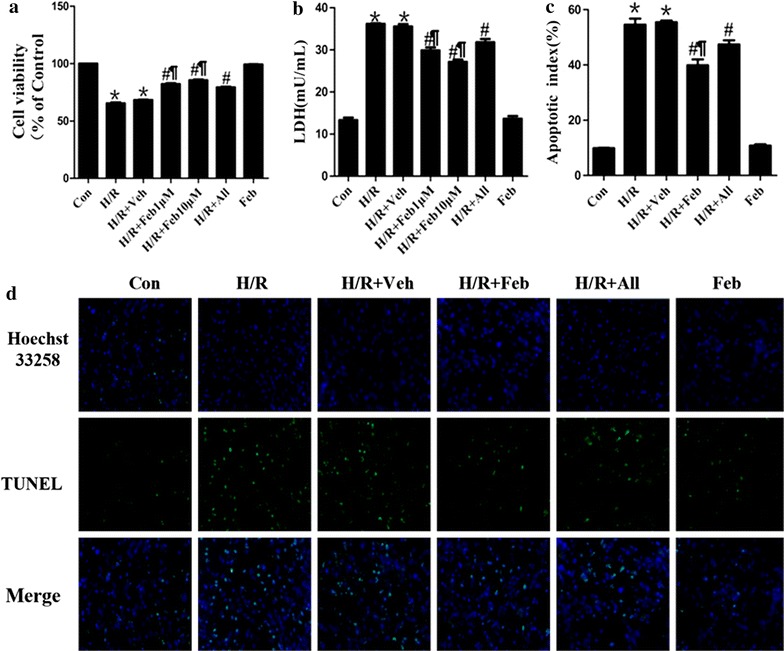
Effects of febuxostat on H/R injury in NRCs. **a** MTT assay following H/R. **b** Leakage of LDH in culture medium. **c** Apoptotic index in NRCs. **d** Typical morphological changes in apoptosis under fluorescence microscope (×200). All values indicate mean ± SD. *P < 0.01 vs. Sham, ^#^ *P* < 0.01, vs. I/R, ^¶^ *P* < 0.01 vs. H/R + All. These experiments were performed in quintuplicate with similar results.

Figure 3a shows viability of NRCs following H/R as detected by an MTT assay Compared with Control group, The viability of H/R and H/R + Veh cells was significantly reduced (65.5 ± 1.3 vs. 100%; 66.6 ± 2.2 vs. 100%, *P* < 0.01, respectively). Pretreatment with febuxostat and allopurinol significantly alleviated this effect (Febuxostat: 80.0 ± 2.7 vs. 65.5 ± 1.3%; Allopurinol: 77.6 ± 2.3 vs. 65.5 ± 1.3%, *P* < 0.01, respectively), especially 10 µM febuxostat restoring cell survival to 96.8 ± 1.4%.

LDH activity was measured to assess cardiomyocyte injury at the end of reoxygenation. LDH activity of H/R cells was markedly increased compared with controls (36.2 ± 0.3 vs. 13.3 ± 1.0, *P* < 0.01). However, febuxostat and allopurinol treatment significantly inhibited the increase in LDH activity (Febuxostat: 28.5 ± 1.0 vs. 36.2 ± 0.3; Allopurinol: 31.8 ± 1.3 vs. 36.2 ± 0.3, *P* < 0.01, respectively). Compared with H/R + All mice, H/R + Feb markedly decreased LDH activity (*P* < 0.01). There was no significant difference between the Control and Feb groups (*P* > 0.05) (Figure 3b).

The anti-apoptotic effects of febuxostat were detected using the TUNEL assay. Compared with the control, significant increases in the percentage of apoptotic cells were observed in H/R (54.6 ± 3.8 vs. 9.9 ± 0.3, *P* < 0.01). However, febuxostat and allopurinol administration suppressed H/R-induced damage of NRCs (Febuxostat: 38.2 ± 2.3 vs. 54.6 ± 3.8; Allopurinol: 45.7 ± 5.4, *P* < 0.01, respectively). In comparison with H/R + All, 10 µM febuxostat treatment significantly decreased AI (*P* < 0.01) (Figure 3c).

### Effect of febuxostat on mitochondrial integrity

**Figure 4:**
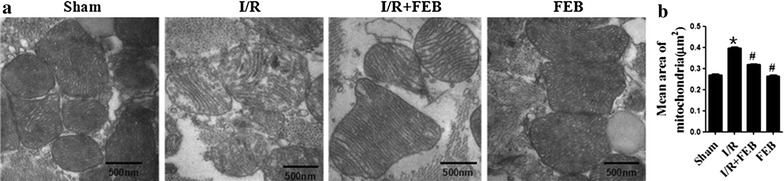
Effect of febuxostat on mitochondrial structure in mice. **a** Representative electron micrographs (×50,000) of mitochondrial structures.* Sham* the structure of mitochondria was generally very good.* I/R* I/R-induced mitochondrial structural disruption (swelling, rupture and loss of cristae).* I/R + FEB* febuxostat treatment ameliorated I/R-induced mitochondrial impairment.* FEB* the structure of mitochondria was basically intact. **b** Mean area of mitochondria. All values represent mean ± SD. *P < 0.01 vs. Sham, ^#^ *P* < 0.01 vs. I/R, n = 4/group, these experiments were performed with similar results.

The mitochondria of Sham mice were regular with unbroken cristae. I/R injury induced mitochondrial swelling and loss of cristae as shown in Figure 3a. However, in the febuxostat- treated group, the outer mitochondrial membranes were intact with regular organization. Febuxostat pretreatment also reduced the degree of mitochondrial swelling. Compared with Sham group, the mitochondrial structure of the FEB group was not significantly altered (Figure 4a). The mean area of mitochondria of I/R group was markedly increased compared with Sham (0.40 ± 0.02 vs. 0.27 ± 0.03, *P* < 0.01). However, febuxostat pretreatment significantly inhibited the increase (0.32 ± 0.02 vs. 0.40 ± 0.02, *P* < 0.01). There was no significant difference between the Sham and FEB groups (*P* > 0.05) (Figure 4b).

### Effect of febuxostat on ROS production and ΔΨm

**Figure 5:**
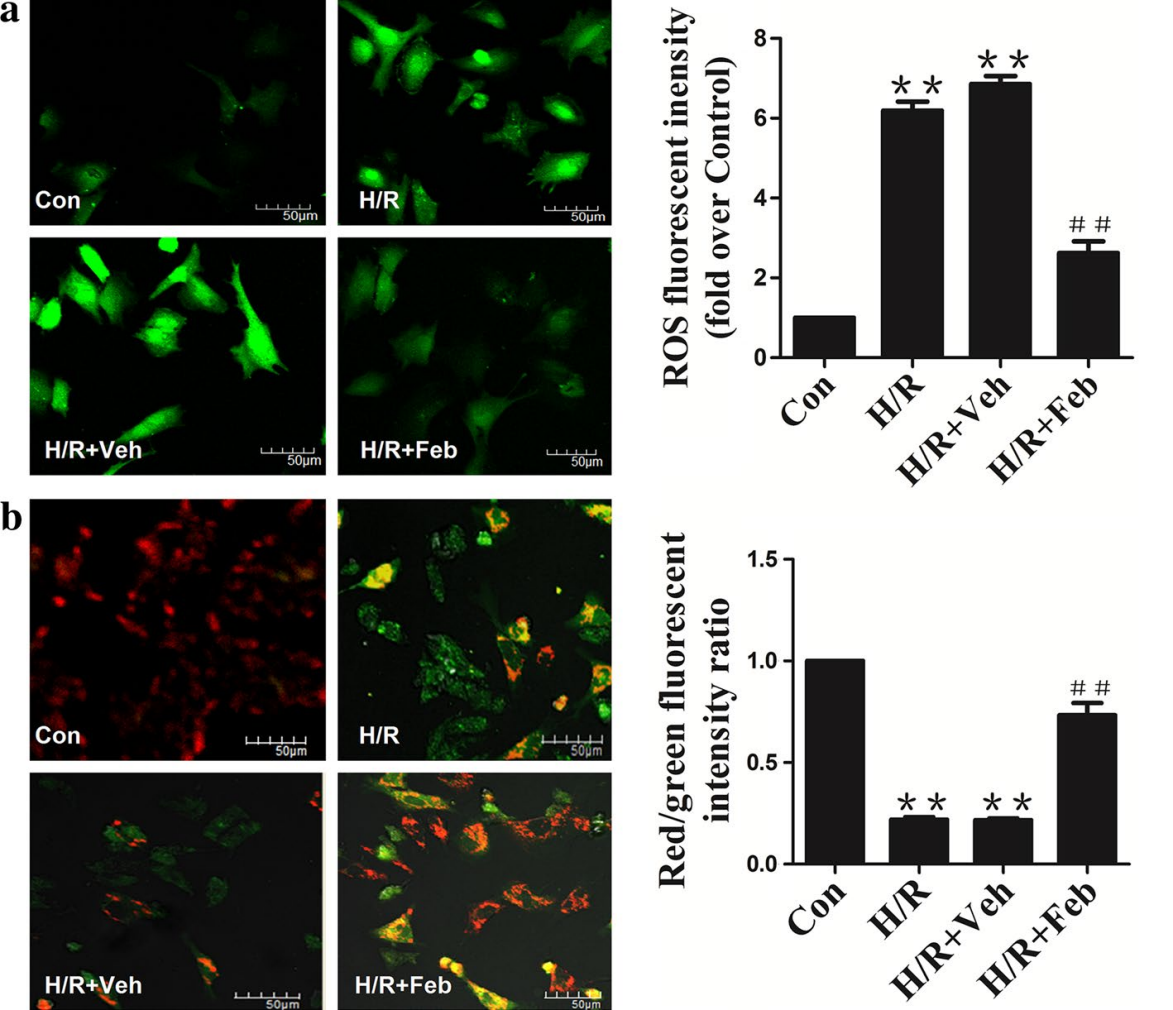
Effect of febuxostat treatment on ROS production and mitochondrial membrane potential (ΔΨm). **a** ROS staining was performed using DCFH-DA. Left: representative images of ROS staining; right: ROS fluorescence intensity. **b** Changes in mitochondrial membrane potential (ΔΨm) are indicated by JC-1 staining; left*: Red fluorescence* represents the mitochondrial aggregate form of JC-1, indicating intact mitochondrial membrane potential. *Green fluorescence* represents the monomeric form of JC-1, indicating dissipation of ΔΨm. Right: ratio of *red*/*green* fluorescence intensity. Both ROS and ΔΨm staining were observed with a laser confocal microscope (×400) (*Bar* 50 µm). All values indicate mean ± SD. **P < 0.01 vs. Con, ^##^ *P* < 0.01 vs. H/R.

We examined the overall ROS generation using the dye DCFH-DA. H/R led to enhanced staining of DCFH-DA, whereas febuxostat treatment decreased staining of DCFH-DA (2.6 ± 0.5 vs. 6.2 ± 0.4, *P* < 0.01) (Figure 5a).

The mitochondrial potential (ΔΨm) was measured in order to investigate mitochondrial function. It was dramatically reduced in H/R and H/R + Veh treated cells. However, febuxostat treatment restored the ΔΨm compared with H/R cells 0.7 ± 0.1 vs. 0.2 ± 0.02, *P* < 0.01) (Figure 5b).

### Effect of febuxostat on cytochrome C, cleaved caspase-3 and -9 expression

**Figure 6:**
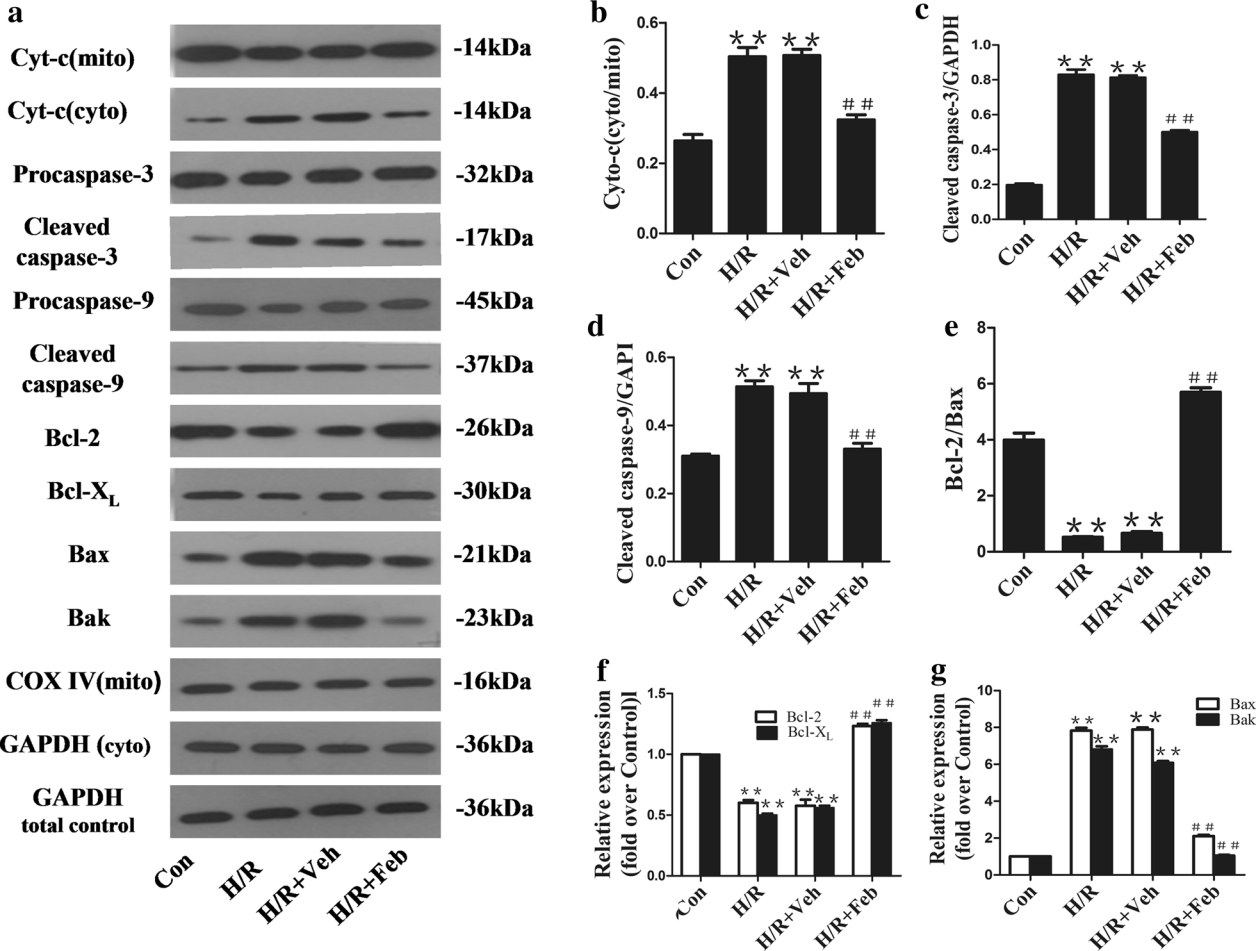
Effect of febuxostat treatment on the expression of proteins regulating mitochondria-mediated apoptosis. **a** Representative Western blots from each group. **b** The levels of Cyto-C (cyto/mito).**c** The levels of cleaved caspase-3. **d** The levels of cleaved caspase-9. **e** The ratio of Bcl-2/Bax. **f** The levels of Bcl-2 and Bcl-X_L_. **g** The levels of Bax and Bak. Levels of proteins were quantified using densitometry and normalized against COX IV or GADPH. Data are presented as mean ± SD. **P < 0.01 vs. Con, ^##^ *P* < 0.01 vs. H/R. These experiments were performed in quintuplicate with similar results.

Western blot analyses indicated that H/R induced a statistically significant increase in cytochrome C released into the cytoplasm compared with the control cells. Febuxostat treatment reduced the release of mitochondrial cytochrome C following H/R injury of NRCs (*P* < 0.01). We also observed the effects of febuxostat treatment on the expression of caspase-3 and -9. There was no significant difference in proprotein expression levels between groups. Cleaved protein expression was significantly increased in H/R cells, while febuxostat treatment reduced the expression of cleaved caspase-3 and -9 (Figure 6a–d).

### Effect of febuxostat on the expression levels of apoptosis-related proteins

In order to investigate whether febuxostat pretreatment protected against H/R-induced apoptosis, we examined the expression of anti-apoptotic (Bcl-2 and Bcl-XL) and pro-apoptotic proteins (Bax and Bak). H/R treatment dramatically reduced Bcl-2 and Bcl-XL, increased Bax and Bak expression and decreased the ratio of Bcl-2/Bax. Following febuxostat treatment, the expression of anti-apoptotic proteins was upregulated, while the level of pro-apoptotic proteins was downregulated. As shown in Figure 6e, the ratio of Bcl-2/Bax was also increased following febuxostat treatment.

## Discussion

The present study yielded several important findings. First, febuxostat pretreatment ameliorated myocardial injury in a mouse I/R model and alleviated H/R injury in cultured NRCs. Second, febuxostat treatment decreased ROS production and inhibited subsequent apoptosis. The underlying protective mechanisms may be attributed to deactivation of a mitochondrial-dependent apoptotic pathway. The role of febuxostat alleviating myocardial ischemia reperfusion injury was superior to allopurinol. This could be due to by higher bioavailability and more potent XO inhibitory effect of febuxostat. Beyond that, febuxostat has fewer side effects than allopurinol.

ROS production is a key mechanism in the injury associated with I/R [18]. During reperfusion, XO is one of the main sources of ROS. XO inhibition with allopurinol modulates ROS production and intracellular Ca^2+^ overload in H/R-injured neonatal rat cardiomyocytes [19]. Treatment with allopurinol has been shown to decrease the infarct areas of myocardial I/R injury in the dog, with XO proposed as the source of free radicals in the myocardium [20]. Febuxostat, a new XO inhibitor, attenuates the pressure overload in LV [21] and protects the kidneys from I/R injury [6] via reduction in ROS production. These observations were consistent with our results that inhibition of XO by febuxostat reduced ROS production.

Together with the reduction of ROS, TUNEL-positive apoptotic cells were also suppressed in febuxostat-treated groups. The possible protective mechanisms of XO inhibitor-induction are mediated by reduced ROS production and mitochondrial protection [22]. This finding is supported by our observation that fubuxostat pretreatment inhibited mitochondrial apoptotic pathway. Hypoxia alters the mitochondrial structure and triggers changes in permeability [23], resulting in functional impairment of the mitochondria, including dissipation of the ΔΨm, release of cytochrome C into the cytoplasm [24] and mitochondrial swelling [25]. Cytochrome C activates caspases, resulting in apoptosis [26, 27]. Our results suggested that the ΔΨm was decreased in H/R cells, but febuxostat restored the Δψm to normal levels. In addition, cytoplasmic cytochrome C and its downstream cleaved caspases were increased in H/R cells, while pretreatment with febuxostat ameliorated these increases. These data demonstrated that cardioprotection by febuxostat was partly accomplished via inhibition of mitochondrial apoptosis.

Febuxostat suppressed apoptosis by another possible mechanism modulated by Bcl-2 family of proteins. Several mitochondrial events are modulated by Bcl-2, especially those linking mitochondrial physiology and apoptosis [28, 29]. Bcl-X_L_ and Bcl-2 (anti-apoptotic proteins) maintain the integrity of the external mitochondrial membrane, preventing the release of cytochrome C from the mitochondria. Conversely, several proapoptotic proteins such as Bax and Bak cause mitochondrial injury, resulting in cell death [30]. In the present study, Western blot revealed that Bcl-X_L_ and Bcl-2 (anti-apoptotic proteins) expression levels were decreased significantly and proapoptotic protein (Bax and Bak) expression was induced by H/R. Febuxostat treatment was also shown to enhance the expression of anti-apoptotic proteins and decrease the expression of proapoptotic proteins, reducing the ratio of Bcl-2/Bax. These results further confirm that febuxostat treatment modulated H/R-induced apoptosis via a mitochondrial-dependent pathway and provide evidence supporting its anti-apoptotic role.

## Conclusion

**Figure 7:**
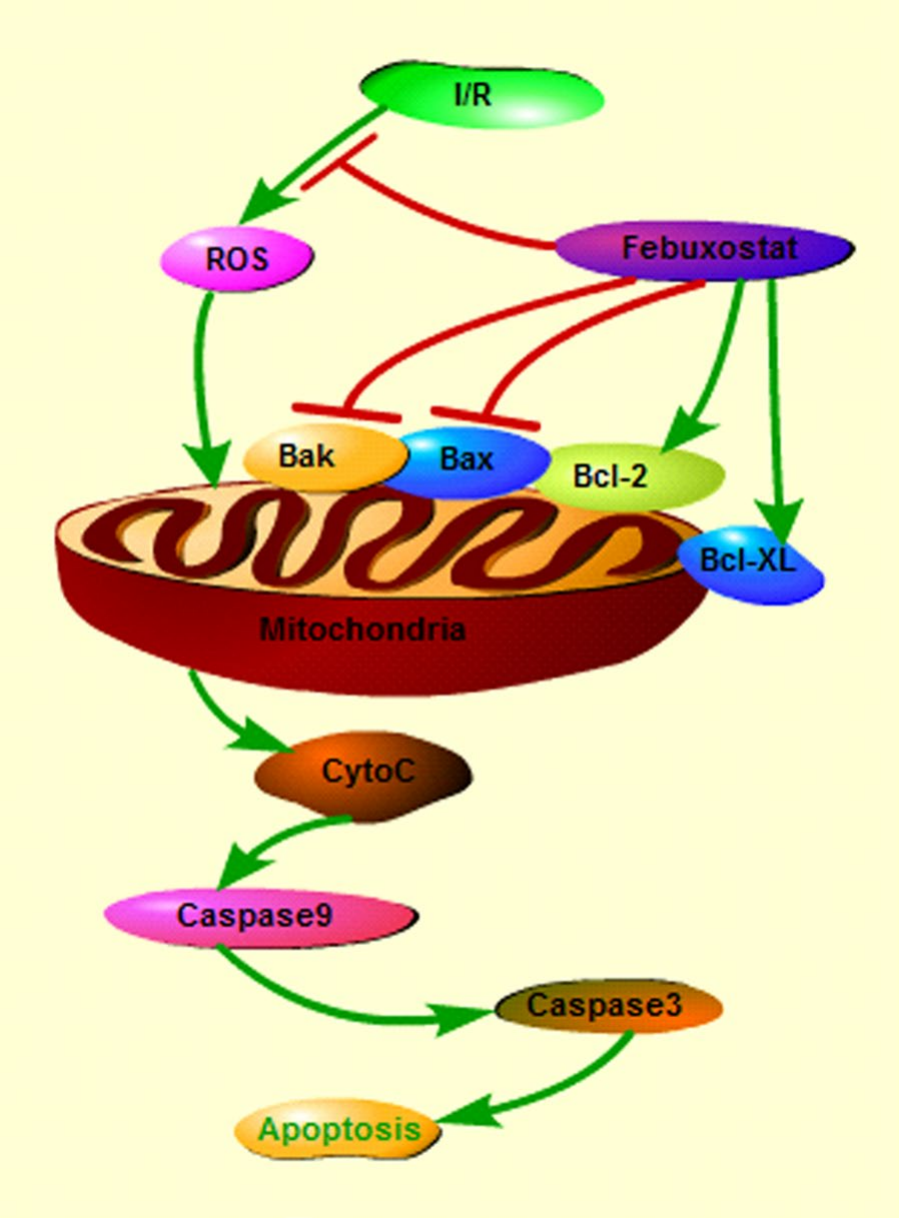
Schematic depicting protective signaling of febuxostat in I/R-induced apoptosis. Febuxostat inhibits the mitochondrial apoptotic pathway by reducing ROS production and modulating Bcl-2 family proteins.

In conclusion, our studies demonstrated for the first time that febuxostat provides cardioprotection following ischemia- and reperfusion-induced myocardial injury by reducing ROS generation and mitochondrial apoptosis (Figure 7). Thus, febuxostat may be a clinically useful agent in myocardial injury.

## Abbreviations

XO: xanthine oxidase
ROS: reactive oxygen species
I/R: ischemia/reperfusion
NRC: neonatal rat cardiomyocyte
H/R: hypoxia/reoxygenation
CK: creatine kinase
LDH: lactate dehydrogenase
AI: apoptotic index
FBS: fetal bovine serum
TTC: 2,3,5-triphenyltetrazolium chloride
DMEM: dulbecco modified eagle’s medium
LAD: left anterior descending
INF: infarct area
AAR: area at risk
LV: left ventricle
LVEDD: left ventricular end diastolic dimension
LVESD: left ventricular end systolic dimension
LVEF: left ventricular ejection fraction
FS: fractional shortening
